# Plant-based meat and dairy alternatives in Norway: consumption, nutritional quality and Nutri-Score based on data from the Norkost 4 survey

**DOI:** 10.1007/s00394-026-04144-y

**Published:** 2026-10-28

**Authors:** Anna Amberntsson, Dina Moxness Konglevoll, Annika Eliassen, Mari Mohn Paulsen, Anne Lise Brantsæter, Marianne Hope Abel, Lene Frost Andersen

**Affiliations:** 1 Department of Food Safety, Norwegian Institute of Public Health, Oslo, Norway; 2 Centre for Sustainable Diets, Norwegian Institute of Public Health, Oslo, Norway; 3 Department of Nutrition, Institute of Basic Medical Sciences, University of Oslo, Oslo, Norway; 4 Faculty of Biosciences, Norwegian University of Life Science, Ås, Norway; 5 Centre for Evaluation of Public Health Measures, Norwegian Institute of Public Health, Oslo, Norway; 6 Department of Physical Health and Ageing, Norwegian Institute of Public Health, Oslo, Norway

**Keywords:** Nutrient profiling, Nutri-Score, Food Rating system, Nutritional quality, Plant-based, Meat and dairy replacement

## Abstract

**Purpose:**

Alternatives to animal-sourced foods (Alt-ASFs) are processed plant-based products that mimic animal-sourced foods. This study aimed to describe the consumption of Alt-ASFs in the Norwegian dietary survey (Norkost 4) and compare population characteristics and nutritional intake in regular consumers. Further, to compare the nutritional quality of Alt-ASFs consumed and describe the distribution of the Nutri-Score and NewTools-score - a proposed revised version of Nutri-Score, among these products.

**Methods:**

Dietary data were collected from 1,815 adults in Norkost 4 using 2*24-h recalls and a food frequency questionnaire. Alt-ASFs were identified and compared with corresponding ASFs. Nutritional quality was assessed and products were scored using Nutri-Score and NewTools-score.

**Results:**

Overall, the consumption of Alt-ASF was low (mean: 0.3 energy %, SD: 1.6). 8% (*n* = 147) reported consuming one or more Alt-ASFs regularly, defined as *≥* 2 times/week, with dairy alternatives being most frequently consumed. Regular consumers were typically younger, female, and more likely to report a vegetarian diet. Most Alt-ASFs contained lower levels of the assessed micronutrients except for meat and fortified milk, whose micronutrient content was similar or higher than ASFs. There was a wide variation in nutritional scores for Alt-ASFs. Unfortified Alt-ASFs typically had a low micronutrient content but could still achieve favourable Nutri-Scores if low in salt, sugar and saturated fat.

**Conclusion:**

Our findings indicate that only a minor part of Norwegian adults regularly consumes Alt-ASFs and consumption is low. Shifting from ASFs to Alt-ASFs may have some nutritional benefits and some disadvantages, largely dependent on the chosen products.

**Supplementary Information:**

The online version contains supplementary material available at https://doi.org/10.1007/s00394-026-04144-y.

## Introduction

In recent years, a global focus on sustainable food systems has intensified [1]. In response to a growing consumer demand for plant-based foods, numerous alternatives to animal-sourced foods (Alt-ASFs) have emerged in the food market [1]. Alt-ASFs refer to processed plant-based products designed to mimic and replace meat, dairy, egg, or fish products [2]. Alt-ASFs offers convenience for the consumers and a pragmatic option for those seeking to eat more plant based [1] or for individuals who cannot consume animal-derived foods because of allergies or intolerances. While these products are often marketed as healthier and more sustainable alternatives to traditional animal-sourced foods (ASF), including meat, dairy, eggs, and fish, their micronutrient content can differ substantially from their corresponding ASFs and whole plant foods [3]. The Alt-ASFs are a heterogeneous group of foods, with diverse nutrient composition largely dependent on the main ingredient [4]. In addition, many Alt-ASFs are highly processed and may contain ingredients of low nutritional quality [5]. Nevertheless, these alternatives represent a food category with a large increase in sales over the past few years and it is predicted that the market will continue to expand globally [6]. Data from Asko, Norway’s largest grocery wholesaler, indicated that the sales in weight of Alt-ASF meat products increased by 244% from 2016 to 2021 [7]. By 2023, this number stagnated, but still represented a 208% increase compared to 2016. However, the annual edible quantity of sold Alt-ASF meat in Norway has been estimated to correspond to approximately 0.2 kg per person in 2023 [7, 8].

The Nutri-Score front-of-pack labelling system has gained traction in several European countries to guide consumers towards healthier food choices by providing a simple, color-coded summary of a product’s nutritional quality [9]. The Nutri-Score algorithm scores food items based on its content of macronutrients as well as content of fruits, vegetables and legumes, which, together with protein, indirectly captures their content of micronutrients. It can be applied to most pre-packed foods and beverages that can be purchased in a store and has a nutritional label [10, 11]. As part of the Norwegian Research Council funded NewTools project [12, 13], minor revisions to the Nutri-Score 2023 algorithms were proposed to better align with the Nordic Nutrition Recommendations 2023 [14] and the Nordic Keyhole label for healthier food options [15, 16]. In this article, we refer to the proposed revised version as the NewTools-score, which is identical to the Nutri-Score for beverages, but gives a different score to approximately 11% of food products in the Norwegian market [15]. Briefly, the algorithm was slightly changed to improve discrimination between whole- and refined grain products and between creams and cheeses with different saturated fat content, to prevent high-sugar products from getting a favorable score, to reward the content of fish, and to automatically assign ‘A’ to raw and unpackaged fruits and vegetables – products that were previously excluded from the algorithm.

Some studies have shown that Alt-ASFs products tend to get a favourable Nutri-Score [4, 17]. While the Nutri-Score has been shown to align well with food-based dietary guidelines in several European countries [18], there is a need for more knowledge on whether the Nutri-Score discriminate the nutritional quality within the Alt-ASFs category. Concern has been raised that the Nutri-Score may contribute to favour the Alt-ASFs over the animal-based counterparts, despite potentially being more highly processed and less nutrient dense [17]. Because the Nutri-Score primarily captures selected macronutrient-related characteristics and only indirectly reflects micronutrient content, it may not fully represent the overall nutritional quality of Alt-ASFs. In an evaluation of Nutri-Score and NewTools-score in a Norwegian setting, using a reference standard based on nutrition experts’ ranking of foods’ healthiness, large disagreements were observed for several plant-based dairy and meat substitutes [19]. In these cases, Nutri‑Score and the NewTools-score generally rated the products as healthier than the nutrition experts did [19].

As the market for Alt-ASFs will potentially continue to expand, it is critical to assess whether labelling systems, such as the Nutri-Score, provide consumers with reliable information on the healthiness of Alt-ASFs to enable informed dietary choices. This study aimed to describe the consumption of Alt-ASFs in the Norwegian dietary survey among adults (Norkost 4) and compare the population characteristics and nutritional intake in regular consumers of Alt-ASF (≥ 2 times per week) with non-regular consumers. We also aimed to compare the nutritional quality of Alt-ASFs consumed in Norkost 4 with their animal-based counterparts. Additionally, we describe the distribution of the Nutri-Score and the NewTools-score for Alt-ASF and ASFs.

## Methods

### Dietary data

Dietary data was derived from Norkost 4, a Norwegian national dietary survey among adults conducted in 2022–2023 [20]. Dietary information was collected from a nationally representative sample (*N* = 1,964) of men (47%) and women (53%) aged 18 to 80 years. Participants completed two randomly distributed 24-h dietary recalls. The participants were contacted by phone by trained study staff, asking to report the full food and beverage intake from the previous day. Following the dietary interviews, participants were asked to answer an online food frequency questionnaire (FFQ) assessing their average consumption of a selection of foods over the past year. Most of the questions in the FFQ were about foods that are not eaten very often, such as Alt-ASFs, and difficult to capture from two 24-h dietary recalls. The participants reported frequency of consumption of the following Alt-ASFs in the FFQ: plant-based sausages or meatballs, plant-based burgers, plant-based meat/soy meat, vegan cheese, and plant-based dairy alternatives (e.g., oat milk, rice milk, soy milk). The pre-defined answer alternatives were: ‘never’, ‘1–2 times per year’, ‘3–11 times per year’, ‘1–3 times per month’, ‘1 time per week’, and ‘≥2times per week’. Participants were also asked to report awareness of a healthy diet (“How much importance do you place on having a healthy diet?”), if they avoided foods because of allergy or intolerance, and if they considered themselves as lacto-ovo-vegetarian, vegan, or flexitarian. Further, the participants also answered questions regarding background information on body weight, height, education level, smoking status, physical activity and use of dietary supplements.

To estimate nutrient content of the food items from the 24-h recalls, the food and nutrient calculation system at the University of Oslo - ‘NutriFoodCalc’ [21] was used, which is based on the 2021 version of the Norwegian food composition Table [22]. The specific database used in this study was from 2022 and included 4,836 food items, although mostly generic foods and only a limited number of Alt-ASF products. Foods that were not reported with their specific product name, or in cases where this specific product did not exist in the database, were registered as the most similar food.

In the current study, participants were excluded if either the 24-h dietary recalls or the FFQ were unanswered (*n* = 149), leaving a final study sample of *n* = 1,815 participants. Consumers and frequency of consumption of Alt-ASFs were identified from the FFQ, while the results from the 2*24-h recalls were used for the description of food intake and nutritional analyses. The Alt-ASFs and ASFs reported in the 24-h recalls were further used for the analysis of nutritional quality of the Alt-ASF products and the corresponding ASF products, and calculation of the Nutri-Score and NewTools-score.

### Alternatives to animal-sourced foods (Alt-ASFs)

As proposed by Qaim et al. [2], we defined the Alt-ASFs as processed plant-based products that try to mimic and replace meat, dairy, egg, or fish. These meat, dairy, egg, or fish analogues are often produced with multiple ingredients and sophisticated processing technologies. Examples include plant-based burger patties, sausages, or plant-based milk and cheese alternatives [2]. Traditional plant-based foods, such as tofu, falafel and margarine, were not included in the definition. Further, plant-based ice cream and vanilla sauce were also excluded as these are considered miscellaneous foods and not as significant contributors to Alt-ASF intake.

All Alt-ASF products identified in the 24-h recalls were included in the nutritional analyses and in the calculation of the Nutri-Score and NewTools-score. The Alt-ASF products included in the analyses corresponded to the specific products registered in the Norkost 4 food database. In total, 41 unique Alt-ASF products were identified and classified into the following Alt-ASF categories: cheese, yoghurt, creams, unfortified milk, fortified milk, flavoured milk, meats and egg alternatives. Each Alt-ASF category was matched to a corresponding ASF category for comparison of nutritional quality and nutritional scores.

### Animal-sourced foods (ASFs)

ASF categories were constructed to match the Alt-ASFs categories. Relevant ASFs were identified from food items reported in the 24-h recalls, excluding home-made mixed dishes. This decision was made to ensure comparability with the Alt-ASF categories, which consisted exclusively of commercially available products rather than composite meals, and since home-made dishes are not covered by the nutritional score algorithms. To create representative ASF comparison groups, the identified ASFs were ranked according to total consumed weight in the Norkost 4 population within each category. The products accounting for 80% of the consumed volume within each category were selected for inclusion. For example, the milk category consists of whole, skimmed and semi-skimmed milk from the most common Norwegian brands while less commonly consumed alternatives like buttermilk and lactose-free milk were not included. Finally, all foods within each category were weighted by consumed weight to balance how much each included food item should account for in the estimated average nutritional content. This approach was used to compare all identified Alt-ASF products with ASF categories that reflected the products most commonly consumed in the study population.

### Calculation of the Nutri-Score and New Tools-score

The Nutri-Score 2023 was calculated for all Alt-ASFs (*n* = 41) and counterpart ASFs (*n* = 90) in the dataset (*N* = 131), using the three algorithms: the main algorithm for general foods, the algorithm for fats, oils, nuts, and seeds, and the algorithm for beverages [10, 11]. The Nutri-Score is calculated based on the content per 100 g of the following unfavourable components: energy (kJ), saturated fat (g), salt (g), total sugar (g), and for beverages, also the presence of non-nutritive sweeteners, and the following favourable components: protein (g), fibre (g), and fruits, vegetables, and legumes (%). No food item assessed contained more than 40% fruits, vegetables and legumes and thus, none got points for this indicator. The final Nutri-Score is based on algorithm specific cut-offs, defining the A*–*E classification where A represents the highest nutritional quality and E the lowest nutritional quality [10, 11]. To calculate the NewTools-score, the same variables plus variables for the content of fish and total carbohydrates were used [15].

### Statistical analysis

Regular consumers of Alt-ASFs were defined as those having a consumption of ≥ 2 times per week of any of the Alt-ASFs.

Frequency of consumed Alt-ASFs, and background characteristics, and overall nutritional intake among regular and non-regular consumers of Alt-ASFs were presented using descriptive statistics. Differences in background characteristics and overall nutritional intake between regular and non-regular Alt-ASFs consumers were assessed using Mann-Whitney U-test for continuous variables and Chi-2 test or Fisher’s exact test for categorical variables.

For the nutritional analyses, the nutrients examined were chosen because animal foods are important sources of them and based on their importance to health [14]. In addition to the macronutrients, the following micronutrients were investigated: salt, vitamin D and B12, folate, calcium, iron, zinc, selenium and iodine. This nutrient selection was guided by a replacement perspective rather than a comprehensive assessment of all potentially beneficial nutrients in plant-based foods, to assess the extent to which Alt-ASFs nutritionally replace the ASFs they are intended to substitute. The percentage of nutritional contribution to the Norwegian Reference values for energy and nutrients [23] (which is based on the Nordic Nutrition Recommendations 2023 [14]) per serving of Alt-ASFs and ASFs was calculated. The calculation was based on the percentage of daily reference value (energy), recommended intake (fat, protein, carbohydrates, fibre, iron, vitamin D and B12, iron, calcium, folate, iodine, selenium, and zinc) and maximum recommended intake (saturated fat, added sugar, and salt).

The discriminatory ability of the Nutri-Score and the NewTools-score within Alt-ASFs and ASFs was assessed using bar charts.

The statistical analyses were conducted in Stata (StataCorp Stata Statistical Software: Release 17, College Station, TX, USA) and statistical significance was set at *p* < 0.05. Figures were created in Stata and Microsoft Excel.

## Results

### Consumption of Alt-ASFs in the Norwegian dietary survey among adults (Norkost 4)

**Fig. 1:**
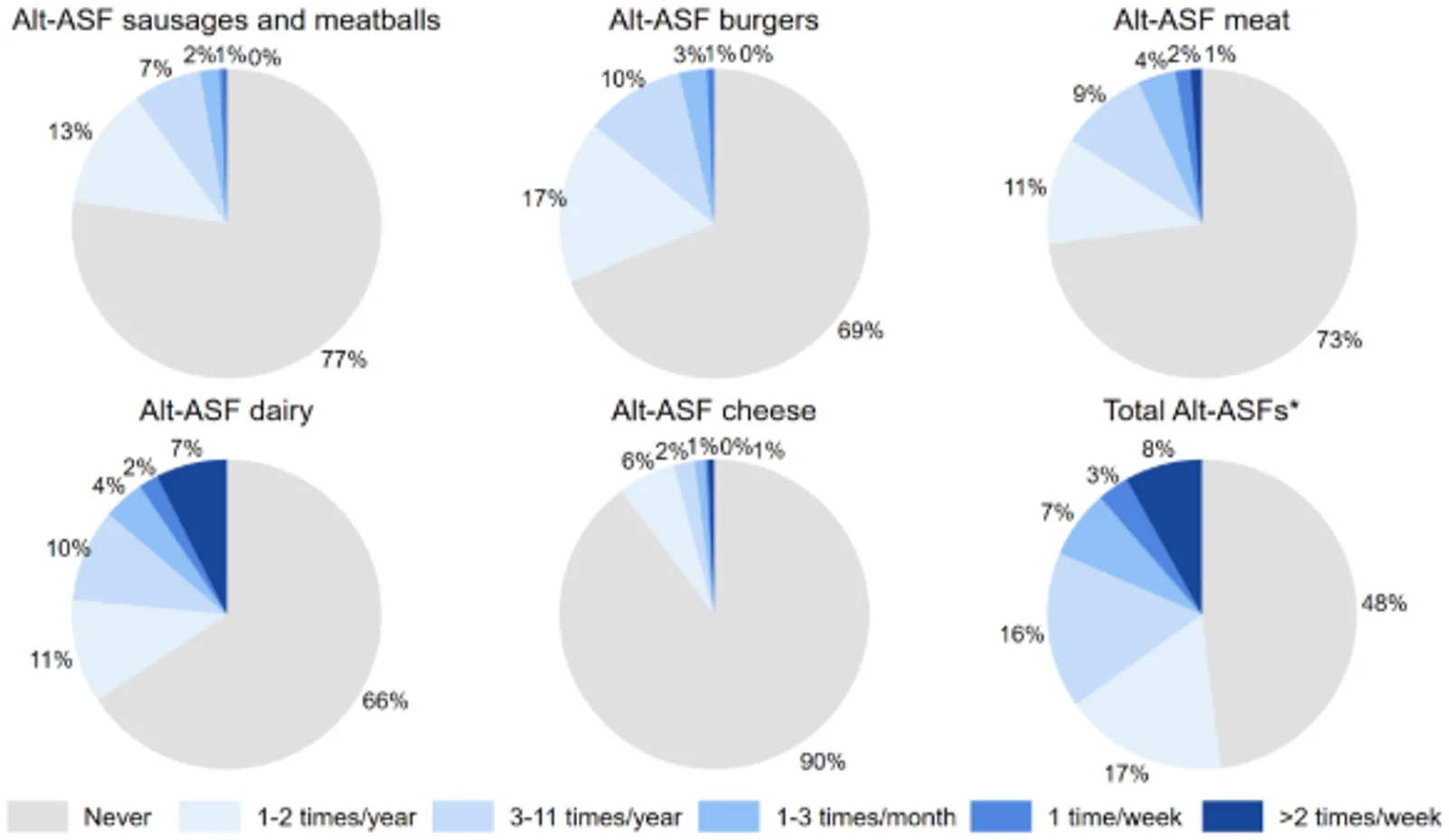
Frequency of consumption of plant-based alternatives to animal-sourced foods (Alt-ASFs) among participants in the Norkost 4 answering the FFQ (*n* = 1,815) Alt-ASFs: Plant-based alternatives to animal-sourced foods. *Highest reported frequency for all five categories of Alt-ASFs

Of the participants in Norkost 4, 1,815 (92%) responded to the FFQ. In the context of this study, these will be referred to as ‘all participants’. Of these, 52% reported having consumed Alt-ASF at least one time in the past year. Monthly, or more often consumption of at least one category of Alt-ASF was reported by 19%, and weekly consumption (≥ 1 time/week) by 11% of all participants. Regular Alt-ASF consumption (≥ 2 times/week) was reported by 8.1%. The most frequently reported Alt-ASF category in the FFQ was dairy alternatives, where 7.4% reported consumption ≥ 2 times/week, followed by meat (1.2%) and cheese alternatives (0.6%, Fig. 1).

### Population characteristics and nutritional intake among regular and non-regular consumers of Alt-ASFs

Compared with non-regular consumers, the regular Alt-ASF consumers comprised a larger proportion of women, were younger, and had a higher education and a lower BMI (Table 1). There was no difference in the proportion of participants who were smokers or in physical activity behaviour between consumers and non-consumers. The regular Alt-ASFs consumers more frequently reported to have a high or very high healthy diet awareness, to use dietary supplements, and to have a vegan or vegetarian diet compared with non-regular consumers. There was also a higher proportion reporting allergies or intolerance with lactose and dairy products among the regular than the non-regular consumers.

**Table 1:** Self-reported characteristics among regular and non-regular consumers of Alt-ASFs among participants in the Norkost 4 answering the FFQ (*n* = 1,815)

	All participants *N* (%)	Non-regular consumers ^a^ *N* (%)	Regular consumers ^a^ *N* (%)	*P*-value ^b^
Participants	1,815 (100)	1,668 (92)	147 (8)	
Sex, women	989 (54)	878 (53)	111 (76)	< 0.001
Age, years	46 (32–59)	47 (33–60)	38 (29–52)	< 0.001
Education, high ^c^	1,102 (61)	1,000 (60)	102 (69)	0.025
Smoking				0.061
Never	1,634 (90)	1,503 (90)	131 (89)	
Sometimes	56 (3)	47 (3)	9 (6)	
Daily	120 (7)	113 (7)	7 (5)	
Physical activity ^c^				0.458
Sedentary	177 (10)	167 (10)	10 (7)	
Moderately active	858 (47)	786 (47)	72 (49)	
Active	618 (34)	570 (34)	48 (33)	
Very active	156 (9)	140 (9)	16 (11)	
Dietary supplement use	1,049 (58)	944 (57)	105 (71)	< 0.001
Healthy diet awareness				< 0.001
None/little/some	786 (44)	750 (45)	36 (25)	
Much/very much	998 (56)	889 (55)	109 (75)	
Lactose intolerance	137 (8)	107 (6)	30 (20)	< 0.001
Dairy product allergy/intolerance	65 (4)	37 (2)	28 (19)	< 0.001
Diet preference				0.011
Vegan	7 (0.4)	1 (0.06)	6 (4)	
Lacto-ovo-vegetarian	20 (1)	9 (0.5)	11 (7)	
Flexitarian	37 (2)	26 (2)	11 (7)	
BMI, kg/m^2^				
Median (p25-p75)	*24.8 (22.5–27.8)*	*24.9 (22.6–27.8)*	*23.9 (21.5–26.8)*	0.002

*FFQ *Food frequency questionnaire, *Alt-ASFs* Plant-based alternatives to animal-sourced foods, *p25-p75* The 25th and 75th percentiles

a Non-regular consumers: *≤*1 times week. Regular consumers: *≥*2 times per week

^**b**^ Testing statistical difference between regular consumers and non-consumers of Alt-ASF using Mann-Whitney U-test for continuous variables and Chi-2 test or Fisher’s exact test for categorical variables

c University/college

d Sedentary’: mainly reading, sitting by the PC/TV, or other sedentary activity, ‘Moderately active’: walking, cycling, or other forms of exercise at least 4 h per week (includes transport to/from work, Sunday strolls etc.), ‘Active’: participation in recreational sports, heavy outside activity, shovelling snow etc. at least 4 h per week, ‘Very active’: participation in hard training or sports competitions regularly several times a week

In all groups, median daily intake of all Alt-ASF categories was 0 g (Table 2). The only exception was median intake of Alt-ASF milk among regular consumers, which was 65 g/day. Considering average intakes, mean intake of all Alt-ASFs were significantly higher in regular consumers than in non-regular consumers. Regarding ASFs, intake of meat, total dairy and milk was higher in non-regular consumers of Alt-ASF. Moreover, regular Alt-ASF consumers obtained more energy from Alt-ASFs and less from ASFs compared with non-regular consumers.

Overall, the consumption of Alt-ASF products was low, contributing on average with 0.3% (SD: 1.6) of the daily energy intake in all participants, and 3.2 E% (SD: 4.2) when restricting to regular Alt-ASF users (Table 2). Among regular consumers, 67% (*n* = 99) reported consumption of Alt-ASFs in the 2*24-h recalls. Among non-regular consumers, 3% (*n* = 49) consumed Alt-ASFs in the dietary recalls.

**Table 2:** Daily mean and median food intake and energy (%) from plant-based alternatives to animal-sourced foods (Alt-ASFs) and ASFs among regular and non-regular consumers of Alt-ASFs among participants in the Norkost 4 answering the FFQ

Alt-ASF (g/day)	All participants (*n* = 1,815)	Non-regular consumers ^a^ (*n* = 1,668)	Regular consumers ^a^ (*n* = 147)	*P*-value ^b^
	Mean (SD) Median (p25-p75)	Mean (SD) Median (p25-p75)	Mean (SD) Median (p25-p75)	
Cheese	0.04 (0.9)	0 (0)	0.5 (3.0)	< 0.001
	0 (0)	0 (0)	0 (0)	
Yoghurt	0.3 (4.5)	0.1 (2.4)	2.4 (13)	< 0.001
	0 (0)	0 (0)	0 (0)	
Creams	0.3 (3.3)	0.1 (1.8)	2.0 (9.7)	< 0.001
	0 (0)	0 (0)	0 (0)	
Milk	9.8 (49)	1.5 (14)	103 (133)	< 0.001
	0 (0)	0 (0)	65 (0–172)	
*Unfortified*	2.5 (25)	0.5 (7.3)	25 (83)	< 0.001
	0 (0)	0 (0)	0 (0–124)	
*Fortified*	7.1 (41)	0.9 (11)	78 (118)	< 0.001
	0 (0)	0 (0)	0 (0)	
*Flavoured*	0.1 (3.1)	0.1 (3.1)	0.3 (3.2)	0.002
	0 (0)	0 (0)	0 (0)	
Nuggets	0.1 (2.8)	0.1 (2.3)	0.5 (6.2)	0.11
	0 (0)	0 (0)	0 (0)	
Burgers	0.2 (4.0)	0.1 (3.5)	1.2 (7.3)	< 0.001
	0 (0)	0 (0)	0 (0)	
Minced meat	0.2 (3.7)	0.1 (2.3)	1.2 (10)	0.08
	0 (0)	0 (0)	0 (0)	
Sausages	0.1 (2.1)	0.02 (0.7)	1.0 (7.0)	< 0.001
	0 (0)	0 (0)	0 (0)	
Meatballs	0.2 (3.3)	0.1 (3.1)	0.6 (5.2)	0.009
	0 (0)	0 (0)	0 (0)	
Egg	0.01 (0.4)	0 (0)	0.1 (1.2)	< 0.001
	0 (0)	0 (0)	0 (0)	
ASF (g/day)				
Cheese	49 (47)	49 (47)	46 (43)	0.23
	39 (18–68)	40 (18–68)	35 (15–68)	
Dairy^d^	245 (249)	254 (246)	129 (154)	< 0.001
	183 (61–345)	193 (71–361)	67 (4–215)	
*Yoghurt*	26 (58)	26 (58)	25 (53)	0.87
	0 (0–9)	0 (0–9)	0 (0–12)	
*Creams*	29 (50)	30 (51)	22 (38)	0.07
	3.7 (0–38)	4 (0–38)	0 (0–31)	
*Milk*	178 (229)	189 (234)	74 (124)	< 0.001
	107 (0–271)	115 (0–289)	0 (0–112)	
Meat, all types	146 (117)	151 (117)	97 (102)	<0.001
	127 (64–198)	131 (71–201)	74 (8.3–151)	
*Red meat* ^c^	103 (104)	106 (106)	67 (76)	< 0.001
	79 (28–147)	82 (30–150)	45 (0–108)	
*White meat* ^c^	40 (67)	41 (67)	30 (59)	0.03
	0 (0–70)	0 (0–71)	0 (0–36)	
Fish and seafood	49 (74)	49 (74)	47 (68)	0.83
	13 (0–77)	13 (0–77)	15 (0–75)	
Eggs	27 (39)	27 (39)	28 (41)	0.65
	0 (0–52)	0 (0–52)	3.5 (0–53)	
Energy from foods (E%)				
Alt-ASF	0.3 (1.6)	0.1 (0.5)	3.2 (4.2)	< 0.001
	0 (0)	0 (0)	1.8 (0–4.5)	
ASF^e^	42 (14)	43 (13)	35 (17)	< 0.001
	41 (33–51)	42 (34–51)	35 (24–45)	

*Alt-ASF* Plant-based alternatives to animal-sourced foods, *SD* Standard deviation, *E% *Energy percentage, *p25-p75* The 25th and 75th percentiles

^a^ Non-regular consumers: *≤*1 times week. Regular consumers: *≥*2 times per week

^b^ Testing statistical difference between regular consumers and non-consumers of Alt-ASF using Mann-Whitney U-test for continuous variables

^c^ Unprocessed and processed meat

^d^ Milk, yoghurt, creams, butter and products. Not cheese

^e^ Meat, egg, fish and seafood, dairy (milk, yoghurt, creams, butter, cheese) and products of these

Table 3 shows that regular consumers of Alt-ASFs overall had slightly lower protein intake than non-regular consumers. Regular Alt-ASF consumers had an overall higher intake of fibre, folate and iron, but lower intake of vitamin B12, zinc, iodine and salt compared with non-regular consumers.

**Table 3:** Nutrient intake from foods among regular and non-regular consumers of Alt-ASFs in the Norkost 4 responders to the FFQ (*n* = 1,815)

Macronutrients	All participants (*n* = 1,815)	Non-regular consumers ^a^ (*n* = 1,668)	Regular consumers ^a^ (*n* = 147)	*P*-value ^b^
	Mean (SD)	Mean (SD)	Mean (SD)	
Energy, MJ	9.1 (3.1)	9.1 (3.1)	8.8 (2.8)	0.15
Protein, E%	18 (4.4)	18 (4.4)	16 (4.4)	< 0.001
Total fat, E%	38 (7.5)	38 (7.4)	38 (8.2)	0.67
Saturated fat, E%	14 (3.9)	14 (3.8)	13 (4.2)	0.11
Carbohydrate, E%	40 (8.3)	40 (8.1)	41 (9.5)	0.23
Added sugar, E%	6.9 (5.4)	7.0 (5.4)	6.4 (5.1)	0.27
Fibre, g	24 (10)	24 (9.9)	28 (12)	< 0.001
Micronutrients				
Vitamin D, µg	5.7 (5.1)	5.7 (5.2)	5.8 (4.6)	0.79
Vitamin C, mg	98 (62)	97 (62)	107 (66)	0.07
Vitamin B12, µg	6.5 (4.5)	6.6 (4.6)	5.2 (2.9)	< 0.001
Folate, µg	261 (102)	258 (100)	285 (114)	0.002
Iron, mg	10 (4.0)	10 (4.0)	11 (4.1)	0.05
Calcium, mg	966 (467)	970 (469)	912 (433)	0.15
Zinc, mg	11 (4.6)	11 (4.6)	10 (3.9)	< 0.001
Selenium, µg	52 (34)	54 (35)	50 (31)	0.26
Iodine, µg	159 (151)	160 (153)	136 (127)	0.06
Salt, g	7.6 (3.4)	7.7 (3.4)	7.1 (3.3)	0.03

*FFQ* Food frequency questionnaire, *SD* Standard deviation, *MJ* Mega joule, *E%* Energy percentage, *mg* Milligrams, *µg* Micrograms. Intake of dietary supplements is not included in the estimations

^**a**^ Non-regular consumers: *≤*1 times week. Regular consumers: *≥*2 times per week

b Testing statistical difference between regular consumers and non-consumers of Alt-ASF using two-sample t-test

### Nutritional quality of the Alt-ASFs consumed

**Fig. 2:**
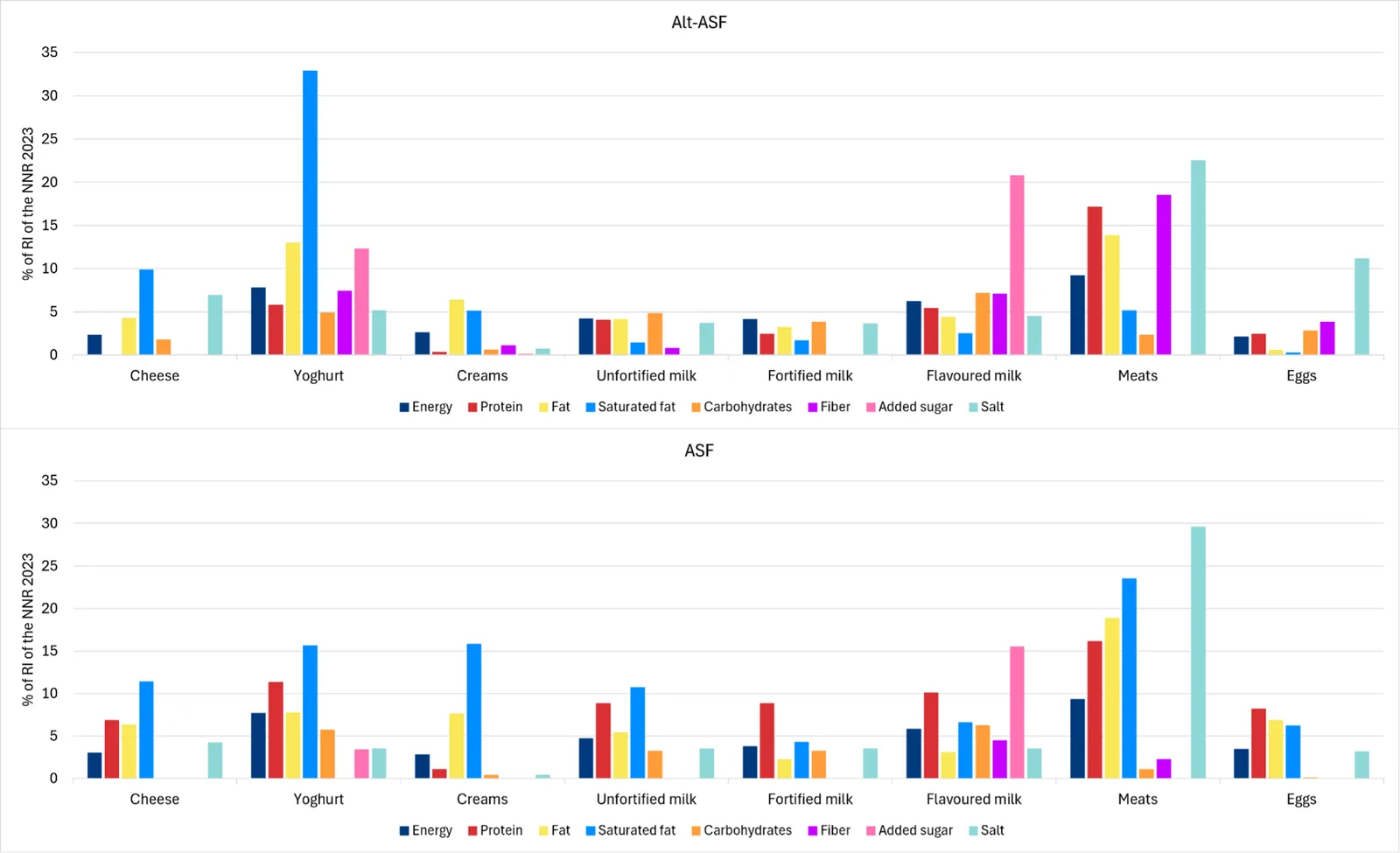
Percentage of recommended intake of macronutrients in the Norwegian Reference values for energy and nutrients and the Nordic Nutrition Recommendations 2023 per serving among food categories of Alt-ASFs (*n* = 41) and ASFs (*n* = 90). Serving sizes: Cheese; 20 g, Yoghurt; 200 g, Creams 30 g, Milk 200 g, Meats 100 g, Eggs 13 g (Alt-ASF) and 80 g (ASF). Alt-ASFs: Plant-based alternatives to animal-sourced foods. ASF: Animal-sourced foods. NNR: Nordic Nutrition Recommendations. RI: Recommended intake

**Fig. 3:**
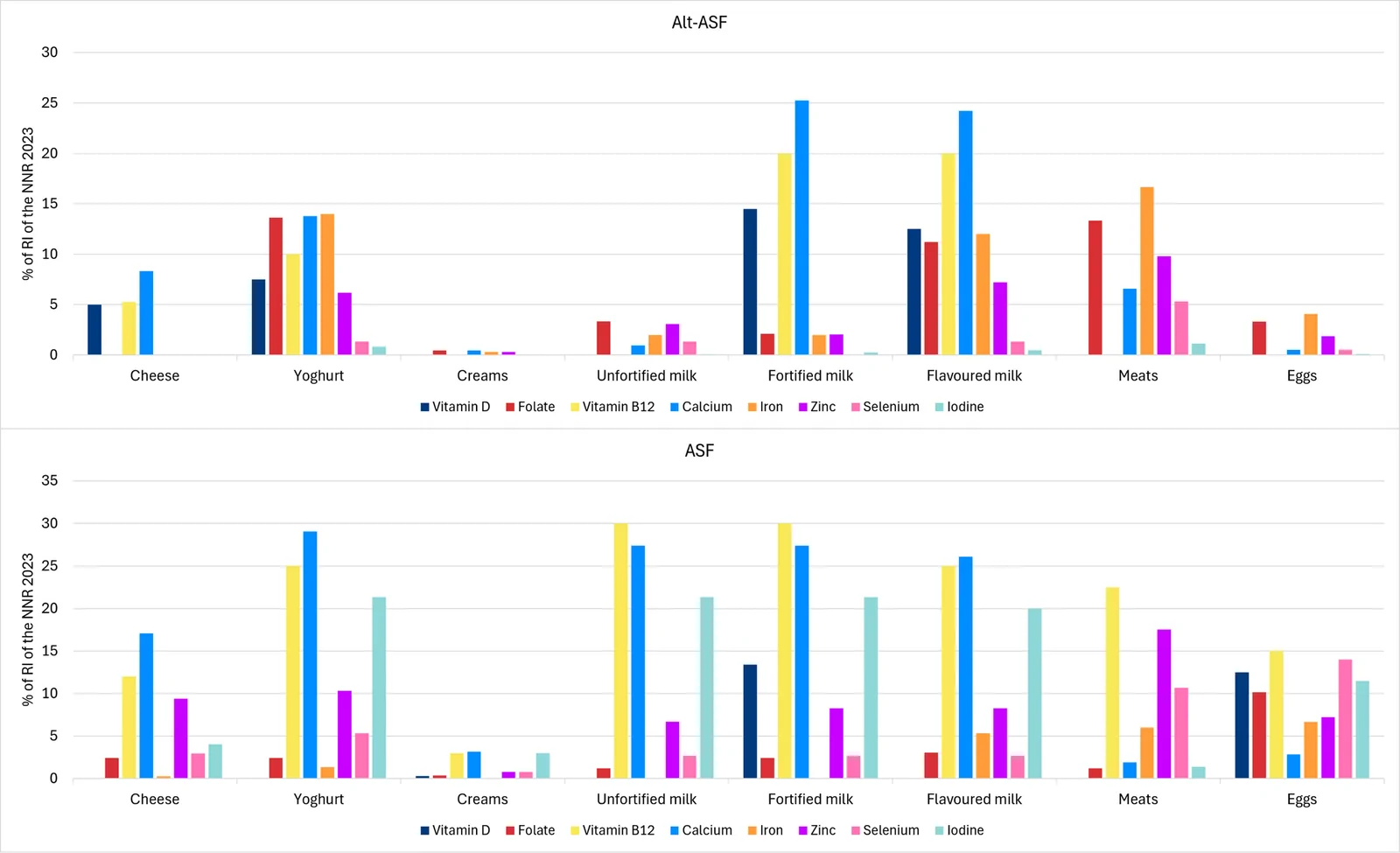
Percentage of recommended intake of micronutrients in the Norwegian Reference values for energy and nutrients [23] and the Nordic Nutrition Recommendations 2023 [14] per serving among food categories of Alt-ASFs (*n* = 41) and ASFs (*n* = 90). Serving sizes: Cheese; 20 g, Yoghurt; 200 g, Creams 30 g, Milk 200 g, Meats 100 g, Eggs 13 g (Alt-ASF) and 80 g (ASF). Alt-ASFs: Plant-based alternatives to animal-sourced foods. ASF: Animal-sourced foods. NNR: Nordic Nutrition Recommendations. RI: Recommended intake

In Norkost 4, there were 41 different Alt-ASF products registered in the database from the 24-h recalls. There was considerable variation in the nutritional content of Alt-ASFs compared with ASFs (Figs. 2 and 3, Supplementary Table 1). Alt-ASF cheese provided substantially lower amounts of protein, zinc, vitamin B12, and calcium, with calcium content reaching only about half of that of the ASF cheese (Figs. 2 and 3). Alt-ASF yoghurt contained markedly more saturated fat — up to one third of the recommended daily maximum — while contributing with considerably less vitamin B12, calcium, and iodine, but more iron. Alt-ASF creams had similar total fat content as that of dairy creams but substantially lower saturated fat levels. Among Alt-ASF milk, nutrient contributions varied by fortification status: unfortified products provided negligible amounts of vitamin D, vitamin B12, and calcium, whereas fortified products (i.e., soy, almond, oat and coconut milk) supplied vitamin D and similar levels of vitamin B12 and calcium as fortified dairy milk. Alt-ASF meat contained less saturated fat and salt but more fibre, folate, and iron, although they provided less vitamin B12 than animal meat. Alt-ASF egg (i.e., egg replacement powder) contributed with minimal nutrients compared with hen’s eggs.

Supplementary Table 2 shows the main ingredient in all Alt-ASFs in the database. Together with fortification, the main ingredient largely affected the nutritional content and variation within the Alt-ASF categories. Alt-ASFs generally contained less protein and fewer micronutrients than ASFs.

### Scoring of Alt-ASFs and ASFs with the Nutri-Score and the NewTools-score

**Fig. 4:**
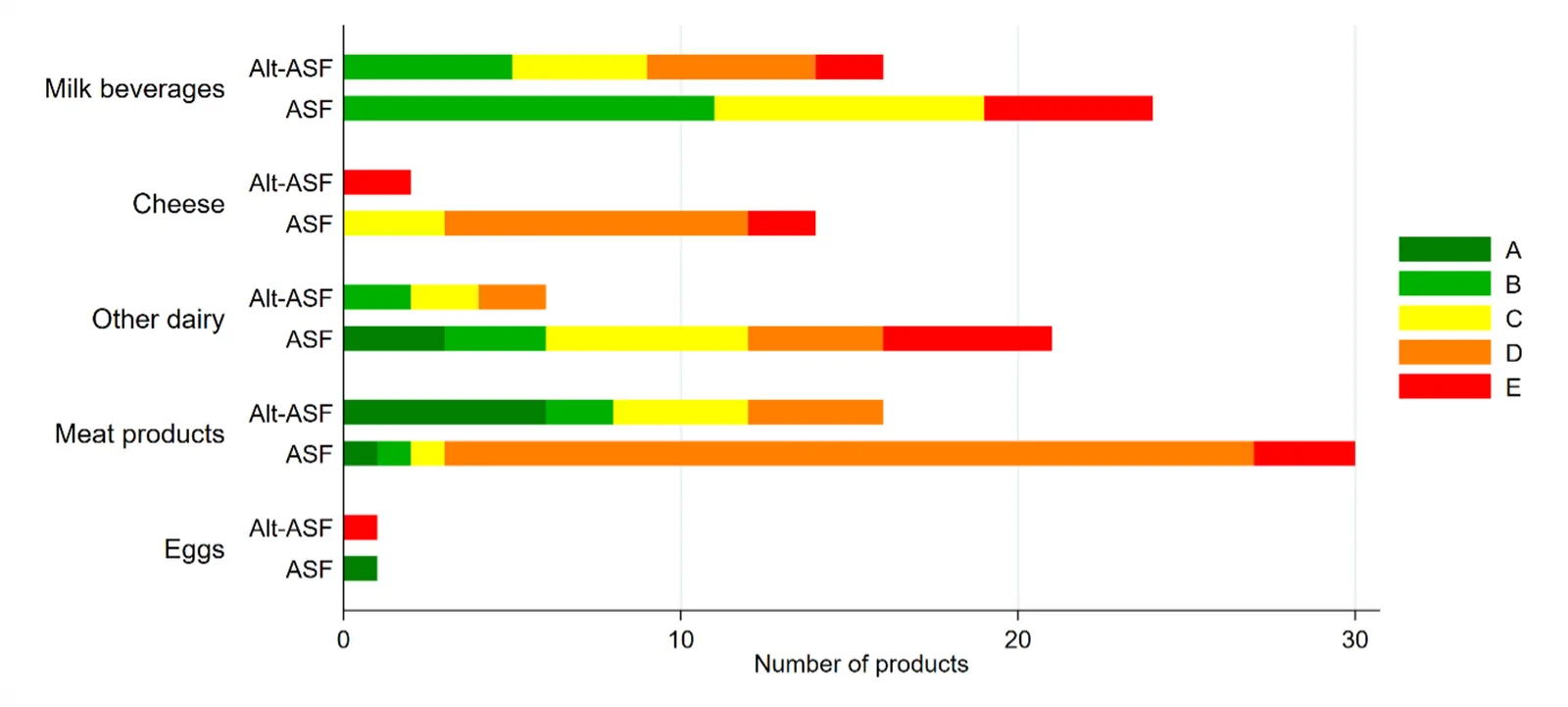
Nutri-Score among Alt-ASFs and ASFs (*n* = 131) consumed in the Norkost 4 dietary survey. Alt-ASFs: Plant-based alternatives to animal-sourced foods. ASF: Animal-scoured foods

The scoring of all Alt-ASFs and ASFs was identical between the Nutri-Score and NewTools-score. Thus, results are only presented for the Nutri-Score. Nutri-Scores varied within several Alt-ASF categories, indicating differences in nutritional composition between products within the same category (Fig. 4). However, some categories contained only a small number of products and should therefore be interpreted with caution. In general, Alt-ASF cheese, yoghurt, milk and eggs got less favourable Nutri-Scores than their ASF counterparts, while Alt-ASF creams and meat generally got a more favourable Nutri-Score. These differences were primarily explained by variations in energy, saturated fat, sugar, salt, and protein content between Alt-ASFs and the corresponding ASF products. More favourable scores among Alt-ASF creams were mainly driven by lower energy and saturated fat content, and for Alt-ASF meat, the scoring was driven by lower saturated fat and salt, and higher fibre content. Less favourable scores among Alt-ASF dairy and egg alternatives were generally related to higher contents of saturated fat, sugar, and/or salt, and lower protein content (Fig. 2 and Supplementary Table 1).

## Discussion

This study provides insights into the consumption and nutritional quality of selected processed plant-based alternatives to animal-sourced foods in the Norwegian diet as captured in the national dietary survey Norkost 4. We found that the overall consumption of the products investigated was very low. Over half of the participants reported having consumed Alt-ASFs at least once in the past year, but only around 8% had consumed these products on a regular basis (*≥* 2 times/week). The category consumed most often was plant-based dairy alternatives. The demographic analysis revealed that regular consumers of Alt-ASFs tended to be younger, predominantly female, higher educated, more often vegetarians, and exhibited increased health awareness compared to non-regular consumers. Most Alt-ASF products were lower in micronutrients compared with the ASF products, with some exceptions such as vitamin D in fortified Alt-ASF dairy alternatives and fibre in Alt-ASF meat. Among the available food items, the Nutri-Score and NewTools-score did not consistently assign more favourable scores to either Alt-ASFs or ASFs. This suggests some ability of the scoring systems to discriminate nutritional differences between Alt-ASFs and ASFs. However, the limited number of products should be considered when interpreting these findings. Neither the Nutri-Score nor the NewTools-score captures fortification or micronutrient content, which is a limitation of these scoring systems.

The demographic profile of regular Alt-ASF consumers aligns with previous research suggesting that women and younger consumers more often choose plant-based alternative foods [24, 25]. The regular Alt-ASF consumers also reported higher use of dietary supplements, which may indicate that they are potentially aware of and compensating for nutrient gaps in their diets. In addition, self-reported food allergies and intolerances were also significantly higher among regular consumers, as well as having a vegan or vegetarian diet preference. Thus, health, food intolerances and diet preferences seem to be key drivers of plant-based food consumption, which is in line with previous studies [1, 25, 26].

Overall, few participants in the Norkost 4 study were regular Alt-ASFs consumers, and even among these, their daily intake of Alt-ASFs were relatively low and did not substantially contribute to the total energy intake. Interestingly, although they had a 36% lower mean intake of meat and 61% lower intake of milk, their total diet intake of iron and calcium were not different compared to the non-regular consumers. However, these findings should be interpreted with caution, as nutrient intakes were not adjusted for total energy intake and the groups differed in demographic characteristics, including age and sex.

Plant-based alternatives are often perceived as inherently healthy, despite considerable variation in their nutritional quality. Our nutritional analysis suggested that differences in micronutrient contributions varied by nutrient and product category. The Alt-ASF cheese and yoghurt products investigated appeared to provide lower amounts of calcium, vitamin B12, and iodine, and the yoghurt alternatives had higher saturated fat content than their dairy equivalents. The latter is likely because one of the two Alt-ASF yoghurts was based on coconut extract which is mostly made up of saturated fat. This emphasizes how vulnerable the analyses are to the characteristics of the specific Alt-ASFs included in the study. Another study on plant-based and animal-based products on the Norwegian market did not find higher saturated fat content in plant-based yoghurt [27]. Fortified plant-based milk products provided similar amounts of vitamin D, calcium and vitamin B12, but no iodine, compared with vitamin D-fortified dairy milk. This finding may reflect the potential role of fortification in improving the micronutrient profile of plant-based alternatives, although the small sample size of products prevents firm conclusions. The Alt-ASF meat included in the analysis contained more fibre, folate, and iron, and less salt, but lower vitamin B12 content than ASF meat. Higher fibre content in plant-based meat has also been found in the study on plant-based and animal-based products on the Norwegian market [27]. It should however be noted that the bioavailability of iron and zinc is generally lower in plant-based sources, which may limit the physiological availability of these nutrients despite higher measured content [28, 29]. Moreover, the primary ingredients of Alt-ASF products varied substantially, even within categories. For example, for Alt-ASF sausages, the main ingredient could be soybean, canola oil, or vegetables. This likely influenced their nutrient profiles. Differences in nutrient composition between ASF and alt-ASF are attributable also to whether the plant-based alternatives are fortified. For alt-ASF dairy, this is particularly relevant for calcium, iodine, vitamin B12, and vitamin D. Within the context of the growing transition towards more plant-based diets, our findings highlight the importance of considering nutritional quality when selecting alternatives to ASFs. However, only a small number of Alt-ASF products were included in the Norkost survey, meaning that the results were largely driven by the specific items included in the analysis and cannot be extrapolated to Alt-ASF products existing on the market.

It is important to note that this study focused exclusively on plant-based products developed to mimic animal-sourced foods, such as plant-based meat, dairy, and egg alternatives. Traditional plant-based foods like legumes and tofu were not included in the analysis because they are generally consumed as foods in their own rather than being specifically formulated and marketed as direct substitutes for ASFs. Neither were dishes or home-prepared products made from vegetables, mushrooms, or grains. Therefore, the findings do not represent a comprehensive assessment of the nutritional consequences of shifting toward a more plant-based diet. Rather, they provide insight into a specific subset of processed plant-based products that are increasingly marketed as direct replacements for animal-based foods.

Front-of-pack nutrition labelling is a tool developed to facilitate healthier food choices by providing consumers with easily interpretable nutrition information. The Nutri-Score might influence purchasing decisions by making consumers aware that the nutritional quality differs both within and between ASF and Alt-ASF categories. Recent reviews suggest that front-of-pack nutrition labelling may influence purchasing decisions, although the magnitude and consistency of these effects remain uncertain, and much of the evidence is derived from experimental rather than real-world settings [30, 31].

The Nutri-Score and NewTools-score classified Alt-ASFs and ASFs similarly. Alt-ASF creams and meat products often received more favourable scores compared with the ASF equivalents, reflecting the lower content of saturated fat and salt in many of the Alt-ASF products. Alt-ASF milk got poorer scoring than ASF milk due to higher content of sugar. The categories Alt-ASF cheese and yoghurt were scored less favourably, due to higher content of salt. More research on all the available Alt-ASF products in the Norwegian market is needed to fully determine the usefulness and discriminatory performance of the Nutri-Score in this food category.

The comparison between the nutrient analyses and Nutri-Score classifications highlights that these approaches capture different aspects of nutritional quality. The nutrient analyses suggested that several Alt-ASFs contained lower amounts of micronutrients than their animal-based counterparts, whereas the Nutri-Score primarily reflected differences in saturated fat, sugar, salt and fibre content. Consequently, products with favourable Nutri-Scores did not necessarily provide the same micronutrient contribution as the corresponding ASFs. This discrepancy was particularly apparent for fortified plant-based milk alternatives and some plant-based meat substitutes. These findings suggest that Nutri-Score may be most informative when interpreted alongside information on fortification and micronutrient composition, especially for consumers using Alt-ASFs as direct replacements for animal-sourced foods.

Given the increasing market share of Alt-ASFs and their perceived health benefits, it is important that front-of-pack nutrition labelling systems provide nuanced and reliable information. The Nutri-Score attempt to bridge the knowledge gap by providing simplified nutritional information. Although neither the Nutri-Score nor the NewTools-score systematically favoured Alt-ASFs over ASFs in the present study, both systems are based primarily on macronutrient composition and do not account for micronutrient content or fortification. This limitation is not unique to Nutri-Score. The Nordic Keyhole, which is the front-of-pack nutrition label currently used in Norway, also relies on nutrient-based criteria, although it additionally requires meat and dairy alternatives (excluding beverages) to contain at least 50% vegetables, legumes or whole grains to be eligible for the label [16]. Together, these findings highlight the need for continued evaluation of front-of-pack labelling systems in the context of emerging plant-based replacement products.

Strengths of the current study include the use of a recent, nationally representable data source with fairly high participation rate. The combination of data from 2*24-h recalls and an FFQ give data on the entire diet of the participants, including both macro and micronutrients. Limitations of this study include the small number of products *–* overall and especially in some categories *–* which affects the generalizability of our findings. Further, reliance on self-reported dietary data – both from the FFQ data frequency of consumption of Alt-ASFs, and daily intake reported in the 24-h recall interviews – is a limitation due to recall bias. The 24-h recall is an open dietary assessment method with the potential to capture a wider variety of foods at a detailed level. Nonetheless, not all reported foods were registered with their specific product name in the database but as a similar type of food. Although the Norkost 4 database covers a large number and variety of foods, product-level information for all items would have been preferable for this study. Moreover, only two days’ reported intake is unlikely to reliably capture infrequently consumed items such as Alt-ASFs, so estimated daily intakes may therefore not accurately reflect habitual consumption. Another limitation includes the uncertainty in nutritional content of the Alt-ASF products as some have been analysed while others were estimated in the food composition database. Thus, the nutritional quality of these products might be over- or underestimated. Another limitation is that home-made dishes containing animal-sourced foods were excluded from the analyses. This was necessary to ensure comparability with the Alt-ASF categories, which consisted solely of ready-to-buy products rather than composite meals. Furthermore, the Nutri-Score is intended for products as purchased and are not directly applicable to home-made dishes without first disaggregating recipes into their constituent ingredients. Consequently, the results should be interpreted as reflecting selected ASF product categories rather than total ASF consumption. While this approach may underestimate the overall contribution of ASFs to the diet, it improves the comparability between ASFs and Alt-ASFs.

## Conclusion

In the Norwegian national dietary survey among adults, consumption was low and contributed minimally to the total energy intake, even in regular consumers. Overall, 8% reported to consume Alt-ASFs regularly, with plant-based milk alternatives being the most common. Regular consumers were typically younger, female, had higher education and were more health conscientious, more likely to have a vegan or vegetarian diet preference and more often reported food allergies or intolerances. Most Alt-ASFs had lower micronutrient content than animal-based foods, though fortified milk and plant-based meat showed some exceptions. Nutri-Score and NewTools-score classified Alt-ASFs and ASFs similarly, though neither system accounts for micronutrient content nor fortification. More comprehensive data on available products and improved nutrient databases are needed to better evaluate the nutritional quality of Alt-ASFs and the suitability of front-of-pack labelling for this product category.

## Supplementary Information

Supplementary Material 1.

## Data Availability

The consent given by the participants does not open for storage of data on an individual level in repositories or journals.
