# Supplementary material for: Plant-based meat and dairy alternatives in Norway: consumption, nutritional quality and Nutri-Score based on data from the Norkost 4 survey
Source: Eur J Nutr. 2026 Oct 28;65(8):288. doi: 10.1007/s00394-026-04144-y (PMC13719784; doi:10.1007/s00394-026-04144-y)
Supplement: Supplementary file 1 [file 394_2026_4144_MOESM1_ESM.docx]

# **Plant-based meat and dairy alternatives in Norway: Consumption, nutritional quality and Nutri-Score based on data from the Norkost 4 survey**

Anna Amberntsson ^1,2^, Dina Moxness Konglevoll ^3^, Annika Eliassen ^4^, Mari Mohn Paulsen ^1,2^, Anne Lise Brantsæter ^1,2^, Marianne Hope Abel ^2,5,6^, Lene Frost Andersen ^3^

^1^ Department of Food Safety, Norwegian Institute of Public Health, Oslo, Norway.

^2^ Centre for Sustainable Diets, Norwegian Institute of Public Health, Oslo, Norway.

^3^ Department of Nutrition, Institute of Basic Medical Sciences, University of Oslo, Oslo, Norway.

^4^ Faculty of Biosciences, Norwegian University of Life Science, Ås, Norway.

^5^ Centre for Evaluation of Public Health Measures, Norwegian Institute of Public Health, Oslo, Norway.

^6^ Department of Physical Health and Ageing, Norwegian Institute of Public Health, Oslo, Norway

**Corresponding author**: Dina Moxness Konglevoll, [d.m.konglevoll@medisin.uio.no](mailto:d.m.konglevoll@medisin.uio.no)

**Supplementary Table 1**. Average nutritional content of the plant-based alternatives to animal-sourced foods (Alt-ASF) (n=41) and animal-sourced foods (ASF) (n=91) products per 100 g registered in the Norkost 4 survey database.

| **Food groups** | **Energy** | **Protein** | **Fat** | **Saturated fat** | **Carbohydrates** | **Fiber** | **Added sugar** | **Salt** | **Vitamin D** | **Folate** | **Vitamin B12** | **Calcium** | **Iron** | **Zinc** | **Selenium** | **Iodine** |
| --- | --- | --- | --- | --- | --- | --- | --- | --- | --- | --- | --- | --- | --- | --- | --- | --- |
| Unit | kJ | g | g | g | g | g | g | Mg | Mcg | Mcg | Mcg | Mg | Mg | Mg | Mcg | mcg |
| Cheese |  |  |  |  |  |  |  |  |  |  |  |  |  |  |  |  |
| Alt-ASF (n=3) | 1034 | 0.1 | 17 | 12 | 25 | 0.0 | 0.0 | 1984 | 2.5 | 0.0 | 1.1 | 396 | 0.0 | 0.0 | 0.0 | 0.1 |
| ASF (n=14) | 1346 | 27 | 25 | 14 | 0.0 | 0.0 | 0.0 | 1200 | 0.0 | 40 | 2.4 | 812 | 0.2 | 4.6 | 11 | 30 |
| Yoghurt |  |  |  |  |  |  |  |  |  |  |  |  |  |  |  |  |
| Alt-ASF (n=2) | 350 | 2.3 | 5.1 | 4.0 | 6.8 | 1.0 | 3.3 | 147 | 0.4 | 22.5 | 0.2 | 66 | 1.1 | 0.3 | 0.5 | 0.6 |
| ASF (n=11) | 344 | 4.5 | 3.0 | 1.9 | 8.0 | 0.0 | 0.9 | 100 | 0.0 | 4.0 | 0.5 | 138 | 0.1 | 0.5 | 2.0 | 16 |
| Creams |  |  |  |  |  |  |  |  |  |  |  |  |  |  |  |  |
| Alt-ASF (n=4) | 773 | 0.9 | 17 | 4.1 | 5.4 | 1.0 | 0.2 | 129 | 0.0 | 5.0 | 0.0 | 15 | 0.2 | 0.1 | 0.0 | 0.1 |
| ASF (n=10) | 843 | 2.8 | 20 | 13 | 3.7 | 0.0 | 0.0 | 75 | 0.1 | 4.0 | 0.4 | 100 | 0.0 | 0.3 | 2.0 | 15 |
| Milk, unfortified |  |  |  |  |  |  |  |  |  |  |  |  |  |  |  |  |
| Alt-ASF (n=7) | 189 | 1.6 | 1.6 | 0.2 | 6.8 | 0.1 | 0.0 | 106 | 0.0 | 5.5 | 0.0 | 4.5 | 0.2 | 0.2 | 0.5 | 0.1 |
| ASF (n=8) | 211 | 3.5 | 2.1 | 1.3 | 4.5 | 0.0 | 0.0 | 100 | 0.0 | 2.0 | 0.6 | 130 | 0.0 | 0.3 | 1.0 | 16 |
| Milk, fortified |  |  |  |  |  |  |  |  |  |  |  |  |  |  |  |  |
| Alt-ASF (n=7) | 186 | 1.0 | 1.3 | 0.2 | 5.3 | 0.0 | 0.0 | 104 | 0.7 | 3.5 | 0.4 | 120 | 0.2 | 0.1 | 0.0 | 0.2 |
| ASF (n=3) | 168 | 3.5 | 0.9 | 0.5 | 4.5 | 0.0 | 0.0 | 103 | 0.7 | 4.0 | 0.6 | 130 | 0.0 | 0.4 | 1.0 | 16 |
| Milk, flavoured |  |  |  |  |  |  |  |  |  |  |  |  |  |  |  |  |
| Alt-ASF (n=2) | 278 | 2.2 | 1.7 | 0.3 | 10 | 1.0 | 5.5 | 130 | 0.6 | 19 | 0.4 | 115 | 0.9 | 0.4 | 0.5 | 0.4 |
| ASF (n=13) | 261 | 4.0 | 2.1 | 0.8 | 8.7 | 0.6 | 4.1 | 100 | 0.0 | 5.0 | 0.5 | 124 | 0.4 | 0.4 | 1.0 | 15 |
| Meats |  |  |  |  |  |  |  |  |  |  |  |  |  |  |  |  |
| Alt-ASF (n=18) | 827 | 14 | 11 | 1.3 | 6.5 | 5.0 | 0.0 | 1294 | 0.0 | 44 | 0.0 | 63 | 2.5 | 1.0 | 4.0 | 1.7 |
| ASF (n=31) | 837 | 13 | 15 | 5.7 | 3.0 | 0.6 | 0.0 | 1700 | 0.0 | 4.0 | 0.9 | 18 | 0.9 | 1.7 | 8.0 | 2.1 |
| Egg |  |  |  |  |  |  |  |  |  |  |  |  |  |  |  |  |
| Alt-ASF (n=1) | 1463 | 15 | 3.2 | 0.5 | 61 | 7.9 | 0.0 | 4926 | 0.0 | 84 | 0.0 | 37 | 4.7 | 1.4 | 3.0 | 1.2 |
| ASF (n=1) | 618 | 13 | 11 | 3.0 | 0.3 | 0.0 | 0.0 | 363 | 2.5 | 67 | 1.2 | 54 | 2.0 | 1.4 | 21 | 34 |

kJ: kilojoule, g: grams, mg: milligrams, mcg: micrograms.

**Supplementary Table 2.** Main ingredient (first ingredient except for water) of the plant-based alternatives to animal-sourced foods (Alt-ASF) in products registered in the database reported in the 24-h recalls in the Norkost 4 survey (n=41). When multiple ingredients are listed, this reflects that different products within the same category had different primary ingredients. Only one main ingredient is shown per product.

| **Alt-ASF category** | **Product count** | **Main ingredient (excluding water)** |
| --- | --- | --- |
| Cheese | 2 | Potato starch |
| Yoghurt | 2 | Soybean, coconut milk |
| Creams | 4 | Oat, soybean, coconut oil |
| Unfortified milk | 4 | Oat, soybean, almond |
| Fortified milk | 10 | Oat, soybean, almond, coconut milk, rice |
| Flavoured milk | 2 | Oat, soybean |
| Sausages | 3 | Soybean, canola oil, vegetables |
| Burgers | 4 | Soybean, potato, pea protein |
| Nuggets | 1 | Coconut oil and canola oil |
| Meatballs | 4 | Soybean, potato, canola oil |
| Minced | 4 | Soybean, pea protein |
| Egg | 1 | Chickpea flour |
